# ADHD pathogenesis in the immune, endocrine and nervous systems of juvenile and maturating SHR and WKY rats

**DOI:** 10.1007/s00213-019-5180-0

**Published:** 2019-02-08

**Authors:** Anna Kozłowska, Paweł Wojtacha, Maciej Równiak, Małgorzata Kolenkiewicz, Andrew Chih Wei Huang

**Affiliations:** 1 Department of Human Physiology, School of Medicine, Collegium Medicum, University of Warmia and Mazury in Olsztyn, Warszawska Av, 30, 10-082 Olsztyn, Poland; 2 Department of Industrial and Food Microbiology, Faculty of Food Science, University of Warmia and Mazury in Olsztyn, Plac Cieszyński 1, 10-726 Olsztyn, Poland; 3 Department of Animal Anatomy and Physiology, Faculty of Biology and Biotechnology, University of Warmia and Mazury in Olsztyn, Plac Łódzki 3, 10-727 Olsztyn, Poland; 4 Department of Pathophysiology, School Medicine, Collegium Medicum, University of Warmia and Mazury in Olsztyn, Olsztyn, Warszawska Av, 30, 10-082 Olsztyn, Poland; 5 Department of Psychology, Fo Guang University, Yilan, 26247 Taiwan

**Keywords:** Steroid hormones, Cytokines, Chemokines, Oxidative stress, Spontaneously hypertensive rat, Attention-deficit/hyperactivity disorder

## Abstract

**Rationale:**

Attention-deficit/hyperactivity disorder (ADHD) is one of the most common neurobehavioural disorders with morphological and functional brain abnormalities. However, there is a growing body of evidence that abnormalities in the immune and endocrine systems may also account for the ADHD pathogenesis.

**Objectives:**

To test ADHD pathogenesis in neurological, immune and endocrine systems, this study examined the concentrations of cytokines, chemokines, oxidative stress markers, metabolic parameters, steroid hormones and steroidogenic enzymes in the serum and/or tissues of spontaneously hypertensive rats (SHRs, animal model of ADHD) and Wistar Kyoto rats (WKYs, control animals). Moreover, the volume of the medial prefrontal cortex (mPFC) as well as the density of dopamine 2 (D_2_) receptor-expressing cells and tyrosine hydroxylase (TH)-positive nerve fibres in it was also elucidated.

**Methods:**

Peripheral blood, spleen and adrenal gland samples, as well as brain sections collected on day 35 (juvenile) and day 70 (maturating) from SHRs and WKYs, were processed by ELISA and immunohistochemistry, respectively.

**Results:**

The results show significant increases of serum and/or tissue concentrations of cytokines, chemokines and oxidative stress markers in juvenile SHRs when compared to the age-matched WKYs. These increases were accompanied by a lowered volume of the mPFC and up-regulation of D_2_ in this brain region. In maturating SHRs, the levels of inflammatory and oxidative stress markers were normalised and accompanied by elevated contents of steroid hormones.

**Conclusions:**

Significant elevations of serum and/or tissue contents of cytokines, chemokines and oxidative stress markers as well as volumetric and neurochemical alterations in the mPFC of juvenile SHRs may suggest the cooperation of neurological and immune systems in the ADHD pathogenesis. Elevated levels of steroid hormones in maturating SHRs may be a compensatory effect involved in reducing inflammation and ADHD symptoms.

## Introduction

Attention-deficit/hyperactivity disorder (ADHD) is a chronic neurodevelopmental disorder that causes hyperactivity, impulsive behaviour and attention problems (Nagui 2009). This condition affects primarily children and teenagers, but it also can continue into adulthood (Dulcan 1997; Paris et al. 2016). Additionally, boys are three times more likely than girls to develop ADHD (Gaub and Carlson 1997; Ramtekkar et al. 2010).

The causes of ADHD are not yet completely understood to date. Most studies suggest that this condition could be linked to the abnormalities in the functioning of the dopaminergic, noradrenergic and/or serotonergic systems (Blum et al. 2008). Moreover, these abnormalities might be associated with brain volume deficits observed in patients with ADHD which were found in the prefrontal cortex, striatum and cerebellum (Mostofsky et al. 2002; Fusar-Poli et al. 2012; van Wingen et al. 2013; Wyciszkiewicz et al. 2017). However, recent findings suggest that steroid hormones also may contribute to ADHD pathogenesis (Martel et al. 2009; Roberts 2016; Roberts et al. 2018). For example, it is suggested that prenatal exposure to elevated testosterone levels may increase risk for early ADHD in young boys and girls (Liu et al. 2012; Roberts and Martel 2013). There is also evidence that testosterone may increase dopamine and vesicular monoamine transporter mRNA expression in the substantia nigra and, in this way, may change the dopamine responsivity of the nigrostriatal pathway which is dysfunctional in children with ADHD (Romanos et al. 2010; Purves-Tyson et al. 2014). More precisely, this pathway conveys signals from the substantia nigra to the caudate nucleus and putamen which are involved in motor control (Stanwood and Zigmond 2000). In turn, oestrogens and progesterone appear to modulate extracellular striatal dopamine concentrations in female rats, but not in males (Xiao and Becker 1994). In addition, women with lower oestrogen levels during the entire cycle may be predisposed to higher ADHD symptoms (Roberts 2016). The participation of corticosteroids in the course of ADHD should also be considered as prenatal exposure to these hormones, similar to testosterone, may lead to mental health risks such as ADHD (Khalife et al. 2013). In addition, low levels of these hormones may disturb the activity of the hypothalamic–pituitary–adrenal axis, which is involved in emotion, learning and attention (for review, see Smith 2006). Moreover, as is widely accepted, glucocorticoids might down-regulate a great number of cytokines, such as interleukin (IL)-1β, IL-6, IL-8, IL-12, IL-18 and tumour necrosis factor alpha (TNF-α) as well as chemokines, such as regulated on activation, normal T-cell expressed and secreted (RANTES) and monocyte chemoattractant protein-1 (MCP-1). However, anti-inflammatory cytokines such as IL-10 and transforming growth factor beta (TGF-β), as well as chemokine IP-10, are up-regulated by these hormones (Batuman et al. 1995; Elenkov et al. 1996; Ramírez et al. 1996; Franchimont et al. 1999; Galon et al. 2002; Fukakusa et al. 2005; Wingett et al. 2011; Martino et al. 2012). Interestingly, significantly higher serum levels of IL-6 and IL-10 were recently reported in children with ADHD (Donfrancesco et al. 2016). Moreover, the levels of pro-inflammatory cytokines were correlated with the severity of ADHD symptoms (Oades et al. 2010b). In addition, detectable levels of various cytokines, including IL-2, IL-5, IL-10 and TNF-β, were also reported in the cerebrospinal fluid of children with ADHD (Mittleman et al. 1997). Thus, all this information coincides well with the fact that cytokines might regulate the basal ganglia and play a pivotal role in the dopamine synthesis in the brain, which is, as was described above, implicated in ADHD (Oades et al. 2010a; Felger and Miller 2012). Accordingly, it is conceivable that alterations in the concentration of cytokines may be influential in the pathogenesis of this condition (Oades et al. 2010a). Moreover, administration of IL-1β, IL-2 and IL-6 in rodents reduced dopamine levels in the brain (Zalcman et al. 1994; Anisman et al. 1996), similar to ADHD patients (Blum et al. 2008). Furthermore, both glucocorticoids and cytokines might increase oxidative stress (Almeida et al. 2011) which was recently reported in children with ADHD (Joseph et al. 2015; Sezen et al. 2016).

Taken together, all the findings presented above suggest that the immune, endocrine and nervous systems may cooperate in ADHD pathogenesis. To test this hypothesis, the present study was designed in the following way. All experiments were done using spontaneously hypertensive rats (SHRs) which are considered as a validated animal model of ADHD, and Wistar Kyoto rats (WKYs) served as a control strain (Sagvolden and Johansen 2012). To evaluate the possible influence of the immune and endocrine systems in ADHD pathogenesis, the contents of cytokines, chemokines, oxidative stress markers and metabolic parameters, as well as steroid hormones and steroidogenic enzymes, were compared in SHRs and WKYs in the peripheral blood and/or adrenal and spleen tissues using commercial ELISA kits. To evaluate brain abnormalities reported in ADHD-affected individuals in various studies (Arnsten 2009) and accompanying possible immune and endocrine alterations detected in the present study, the volumes of the medial prefrontal cortex (mPFC) as well as the density of dopamine 2 (D_2_) receptor-expressing cells and nerve fibres containing tyrosine hydroxylase (TH) in it were also compared in SHRs and WKYs using single-labelling immunohistochemistry. The PFC was chosen for investigation because much of the research on ADHD has pointed to weaknesses in the PFC, the most highly evolved of the association cortices (Arnsten 2009). Moreover, imaging studies have demonstrated that patients with ADHD have alterations in the PFC morphology and circuits and demonstrate weaker PFC activation while trying to regulate attention and behaviour (Mostofsky et al. 2002; Arnsten 2009). D_2_ dopamine receptor was selected since there is evidence that it may mediate hyperactivity and the response to psychostimulants in ADHD-affected individuals (Fan et al. 2010). TH was proposed as a marker because this rate-limiting enzyme of the dopamine synthesis is down-regulated in the PFC of SHRs (Viggiano et al. 2004) and human ADHD patients, and dopamine is essential for proper functioning of the PFC (Arnsten 2009). As many of the brain abnormalities were reported in one of the brain hemispheres only (Rubia et al. 1999; Shaw et al. 2009; Silk et al. 2016; Doi and Shinohara 2017), both left and right hemispheres were investigated. Considering that brain abnormalities associated with ADHD manifest in pre-pubertal SHRs (5-week-old) and they often disappear in maturing SHRs (10-week-old) (Hsu et al. 2010), any abnormalities in studied biologically active substances contents due to ADHD should be observed before puberty. In post-pubertal and mature SHRs, hypertension develops (Louis and Howes 1990). Thus, neurological, hormonal and immune abnormalities after puberty (10-week-old animals) should rather be linked with hypertension. Because there is significant male bias (Gaub and Carlson 1997) in ADHD patients, male SHRs were chosen for investigation.

## Material and methods

### Animals

Juvenile (5-week-old) and mature (10-week-old) male SHRs (*n* = 12) and WKYs (*n* = 12) were used in the present study. Both SHRs and WKYs aged 3 weeks were obtained from Charles River (Germany) and transported to the animal house at the Institute of Animal Reproduction and Food Research of the Polish Academy of Sciences (Olsztyn, Poland) where they were kept in sanitised polypropylene cages in pairs or threes to prevent isolation stress. The temperature-controlled (21 ± 1 °C) and ventilated (12–20 exchanges/h) animal room was maintained on a 12/12 h light/dark cycle (lights on from 06:00 to 18:00 h). All animals were fed with a grain mixture (VRF1 diet; Charles River, Germany) and tap water ad libitum. All experiments were carried out in accordance with the European Union Directive for animal experiments (2010/63/EU) and approved by the Local Ethical Commission of the University of Warmia and Mazury in Olsztyn (no. 43/2014). All efforts were made to minimise animal suffering and to use the minimum number of animals necessary to produce reliable scientific data. The strains of rats used in the present study were carefully chosen. Based on behavioural, genetic and neurobiological data, SHRs obtained from Charles River, Germany, are currently the best-validated animal model of ADHD (Sagvolden and Johansen 2012). WKYs are considered a proper control for these SHRs and the ADHD model. Moreover, exactly the same rat strains had extensive behavioural testing in our previous study (Tsai et al. 2017) which proved ADHD symptoms in SHRs in the open field task (increased motor and decreased anxiety behaviours). Thus, exactly the same rat strains were chosen for the experiments in the present study.

### Experimental procedure and tissue processing

Following the habituation phase, all of the SHRs and WKYs were assigned into four groups according to study design: (1) 5-week-old SHRs (*n* = 6; b.w. 111.10–123.38 g); (2) 5-week-old WKYs (*n* = 6; b.w. 111.25–130.96 g); (3) 10-week-old SHRs (*n* = 6; b.w. 254.72–281.38 g) and (4) 10-week-old WKYs (*n* = 6; b.w. 247.33–266.95 g). Later, all rats were given the same tissue processing to label specific target markers.

#### Blood sample preparation

All rats were first deeply anaesthetised with an intraperitoneal injection of Morbital (Biowet, Poland; 50 mg/kg; 133.3 mg/ml of pentobarbital sodium salt and 26.7 mg/ml of pentobarbital). The abdomen was then opened and blood was drawn from the inferior vena cava into EDTA tubes (42110, FL Medical, Poland) (Palombo et al. 2000). Blood samples were always collected from animals between 7:00 a.m. and 8:00 a.m. within less than 3 min to avoid the initiation of pituitary stress response (Vahl et al. 2005).

#### Adrenal gland and spleen preparation

After blood sample collection, a clip was put on the thoracic part of the aorta just above the diaphragm, and the spleen and adrenal gland were carefully dissected from all studied animals. These tissue samples were immediately placed in liquid nitrogen (− 196 °C) for 30 min, and they were then stored at low temperature (− 80 °C) for further analysis.

#### Brain preparations

After collecting blood, adrenal gland and spleen samples, all animals were transcardially perfused with saline (0.9%), followed by 4% paraformaldehyde (pH 7.4; 1040051000, Merck, Germany) in phosphate-buffered saline (PBS; P5493, Sigma Aldrich, Germany). Following perfusion, the brains were carefully dissected from the skulls and post-fixed by immersion in the same fixative for 24 h, washed three times in 0.1 M phosphate buffer (pH = 7.4, 4 °C) and then stored for 3–5 days in graded solutions (10, 20 and 30%) of sucrose (363-117720907, Alchem, Poland) in 1× PBS at 4 °C until they sunk. The brains were then frozen and coronally sectioned at a thickness of 10 μm using a cryostat (HM525 Zeiss, Germany). The sections were stored at − 80 °C until further processing.

### Methods

#### Immunoenzymatic determination (ELISA) of the levels of cytokines, chemokines, oxidative stress markers, steroids hormones, steroidogenic enzymes and metabolic markers in the serum and/or tissues

To determine the concentrations of cytokines, chemokines, oxidative stress markers, steroid hormones and steroidogenic enzymes in the rat serum and/or tissues, commercial ELISA kits were used according to the manufacturer’s instructions (Table 1). The tissues were homogenised in RIPA buffer in 4 °C and were centrifuged with an acceleration of 30,000 × g for 1 h. After this time, the obtained supernatants of tissues were aliquoted and stored at − 80 °C. These supernatants were used for measurements. The absorbance in the ELISA test plate was measured by plate reader TECAN Infinite M200 PRO (Austria) at the wavelength of *λ* = 492 nm.The concentration of cytokines, chemokines and steroid hormones in tissues was measured by BCA method (Pierce USA; Pierce BCA Protein Assay Kit) and presented per milligram of protein.

**Table 1:** List of ELISA kits used for the determination of studied substances’ concentrations in rat serum and/or tissues

	Antigen	ELISA test and catalogue number	Manufacturer, country	Assay range (pg/ml or ng/ml)
1.	Rat IL-1β	Rat IL-1β Mini ABTS ELISA Development Kit 900-M91	Peprotech, USA	63–4000 pg/ml Intra-assay CV < 9% Inter-assay CV < 10%
2.	Rat IL-6	Rat IL-6 Mini ABTS ELISA Development Kit 900-M86	Peprotech, USA	31–2000 pg/ml Intra-assay CV < 9% Inter-assay CV < 10%
3.	Rat TNF-α	Rat TNF-α Mini TMB ELISA Development Kit 900-TM73	Peprotech, USA	47–6000 pg/ml Intra-assay CV < 9% Inter-assay CV < 10%
4.	TGF-β	TGF beta-1 Multispecies Matched Antibody Pair CHC1683	Thermo Fisher Scientific, USA	62.5–4000 pg/ml Intra-assay CV < 6% Inter-assay CV < 5%
5.	Rat MCP-1	Rat MCP-1 (CCL-2) Mini ABTS ELISA Development Kit 900-M59	Peprotech, USA	16–2000 pg/ml Intra-assay CV < 9% Inter-assay CV < 10%
6.	Rat RANTES	Rat RANTES (CCL5) Mini ABTS ELISA Development Kit 900-M72	Peprotech, USA	16–2000 pg/ml Intra-assay CV < 9% Inter-assay CV < 10%
7.	Rat IP-10	Rat IP-10 (CXCL10) Mini ABTS ELISA Development Kit 900-M449	Peprotech, USA	16–1000 pg/ml Intra-assay CV < 9% Inter-assay CV < 10%
8.	Progesterone	Progesterone ELISA EIA-1561	DRG Instruments	0–40 ng/ml Intra-assay CV < 6.42% Inter-assay CV < 6.63%
9.	Corticosterone	Corticosterone ELISA EIA-4164	DRG Instruments	0–240 pmol/ml Intra-assay CV < 3.096% Inter-assay CV < 6.01%
10	Cortisol	Cortisol ELISA EIA-1887	DRG Instruments	0–800 ng/ml Intra-assay CV < 5.63% Inter-assay CV < 6.93%
11.	Rat CYP450	Rat CYP450 (cytochrome P450) ELISA kit ER0888	Fine Test, China	0.625–40 ng/ml Intra-assay CV < 8% Inter-assay CV < 10%
12	Rat HSD3B1	Rat 3 beta-hydroxysteroid dehydrogenase/delta 5→4-isomerase type 1 (HSD3B1) ELISA Kit CSB-EL010781RA	Cusabio, USA	18.75–1200 pg/ml Intra-assay CV < 8% Inter-assay CV < 10%
13.	Rat CYP21A1	Rat CYP21A1 (steroid 21-hydroxylase) ELISA Kit ER1725	Fine Test, China	0.156–10 ng/ml Intra-assay CV < 8% Inter-assay CV < 10%
14.	Rat CYP11B1	Rat CYP11B1 (cytochrome P450 11B1, mitochondria) ELISA Kit ER1724	Fine Test, China	5.625–1000 pg/ml Intra-assay CV < 8% Inter-assay CV < 10%
15.	Rat CYP11B2	Rat cytochrome P450 11B2, mitochondrial (CYP11B2) ELISA Kit CSB-EL006391RA	Cusabio, USA	43.75–2800 pg/ml Intra-assay CV < 8% Inter-assay CV < 10%

#### Determination of malondialdehyde in the spleen (thiobarbituric acid assay)

The concentration of malondialdehyde (MDA) was determined according to the method of Weitner et al. (2016) with modifications. Briefly, tissue supernatant with BHT (buthylohydroxytoluene, antioxidant, Sigma Aldrich, USA) was subjected to deproteinisation by adding 20% trichloroacetic acid (TCA, Avantor, Poland) and centrifugated for 1 h; 100 μl of supernatant was then mixed with an acetic acid solution of thiobarbituric acid (TBA, Sigma Aldrich, USA) and incubated for 1 h at 95 °C. The concentration of MDA was read from a calibration curve (TBA Malondialdehyde Standard, Cayman, USA). The absorbance was read in a spectrophotometer (Perkin Elmer, Lambda 25, Biocompare, USA) at a wavelength of *λ* = 520 nm. The concentration of MDA was presented as picomolar per milligram of whole protein in the supernatant of spleen.

#### Determination of sulfhydryl groups (–SH) in the spleen

The sulfhydryl groups were determined using the Ellman modified method described by Chan and Wasserman (1993). Briefly, 1 ml of 40 mM DTNB solution (5,5′dithiobis(2-nitrobenzoic acid); Sigma Aldrich, USA) was added to the sample (86 mM Tris (Serva, Germany), 90 mM glycine (Serva, Germany), 4 mM EDTA (Sigma Aldrich, USA), 8 M urea (Avantor, Poland), 0.5% SDS (sodium dodecyl sulfate, Serva, Germany), 0.2 M Tris HCl (Trizma base, Sigma Aldrich, USA)), pH 8. Next, 200 μl of the samples were added to 1 ml of DTNB solution. Samples were incubated at room temperature for 30 min. Cysteine was used (Sigma Aldrich, USA) as a standard and the absorbance was measured by a spectrophotometer (Perkin Elmer, Lambda 25, Biocompare, USA) at a wavelength of *λ* = 412 nm. The concentration of –SH groups was measured from a calibration curve based on a cysteine solution in PBS. The concentration of the thiol groups was presented as micromolar per milligram of whole protein in the supernatant of spleen.

#### Determination of fructose and glucose in the spleen

Fructose concentration was determined in the spleen by a modified method described by Messineo and Musarra (1972). This method is specific for fructose (similar sucrose and inulin) without interference with aldohexoses (glucose), aldopentoses and ketopentoses. Glucose concentration was evaluated by a modified glucose oxidase Trinder method Lott and Turner (1975) with further modifications for tissue measurements. The spectrophotometric method was used for the measurement according to the Pointe Scientific set number G7521 (Pointe Scientific, USA) with appropriate modifications. The concentrations of fructose and glucose were presented as microgram per milligram of whole protein in the supernatant of spleen.

#### Immunohistochemistry

Alterations in cytokines, chemokines, oxidative stress markers and/or steroid hormone contents in the serum and tissues may have an influence on various brain functions (Zalcman et al. 1994; Anisman et al. 1996; Oades et al. 2010a; Roberts 2016). However, it is unclear if these changes may also be associated with morphological and neurochemical alterations in the brain of ADHD patients. To answer this question, selected brain sections comprising mPFC from SHRs and WKYs were processed for two routine immunohistochemical techniques: immunoperoxidase labelling using 3,3-diaminobenzidine (DAB) as a chromogen and immunofluorescence. All staining procedures were carried out in humid dark chambers (Immuno Slide Staining Trays, R64001-E, Pyramid Innovation Ltd., UK) and at room temperature.

#### DAB method

Brain sections selected for morphometric and stereological procedures were subjected to the single-labelling DAB method (Dako Liquid DAB + Substrate Chromogen System, K3468, Denmark) which was described in detail in our previous paper (Kozłowska et al. 2018). Briefly, these sections were incubated overnight with a solution of primary antibodies directed towards a neuron-specific nuclear protein NeuN (pan-neuronal marker; Anti-NeuN Antibody, clone A60, MAB377; Merck Millipore, Poland; working dilution 1:1000) and then incubated for 1 h with the solution of secondary antibodies (ImmPRESS™ Universal Reagent Anti-Mouse/Rabbit IgG Peroxidase, MP-7500; Vector Laboratories, Inc., Burlingame, CA, USA; working dilution 1:1). Next, these sections were washed in PBS and incubated for 1 min with DAB substrate–chromogen solution. Finally, stained sections were rinsed in tap water, dehydrated through a graded alcohol series (POCH, Poland), cleaned in xylene and mounted in DPX (DPX Mountain for histology; 44581, Sigma Aldrich, Germany).

#### Immunofluorescence

Brain sections selected for neurochemical evaluations were processed for routine single-immunofluorescence labelling as described previously by Kozłowska et al. (2017) and using primary antisera against either TH (mouse, cat. no. MAB 318, EMD Millipore, USA; working dilution 1:1000) or subtype 2 of dopamine receptor (D_2_; rabbit, cat. no. AB5084P, EMD Millipore USA; working dilution 1:1000). Following subsequent rinsing in PBS (3 × 15 min), the sections were incubated (1 h) with the solution of secondary antibodies (Alexa 488, cat. no. A-11001 or Alexa 568, cat. no. A-11011, Thermo Fisher Scientific, USA; working dilution 1:1000) and then coverslipped with Fluorescent Mounting Medium (cat. no. S3023; Agilent, Denmark).

#### Controls of antibodies’ specificity

The antibody against neuron-specific nuclear protein NeuN used in the immunoenzymatic experiment is an excellent marker for neurons in the central and peripheral nervous systems (Mullen et al. 1992). The specificity of primary antibodies used for the immunofluorescence experiment was tested by pre-absorption tests based on the incubation of sections with an antibody that had been pre-absorbed with synthetic antigen (25 μg of appropriate antigen per 1 ml of corresponding antibody at working dilution). Finally, the specificity of secondary antibodies was controlled by the omission and replacement of all primary antisera by non-immune sera or PBS. A lack of any immunoreaction proved specificity.

#### Volumetric measurements and cell counts

**Fig. 1:**
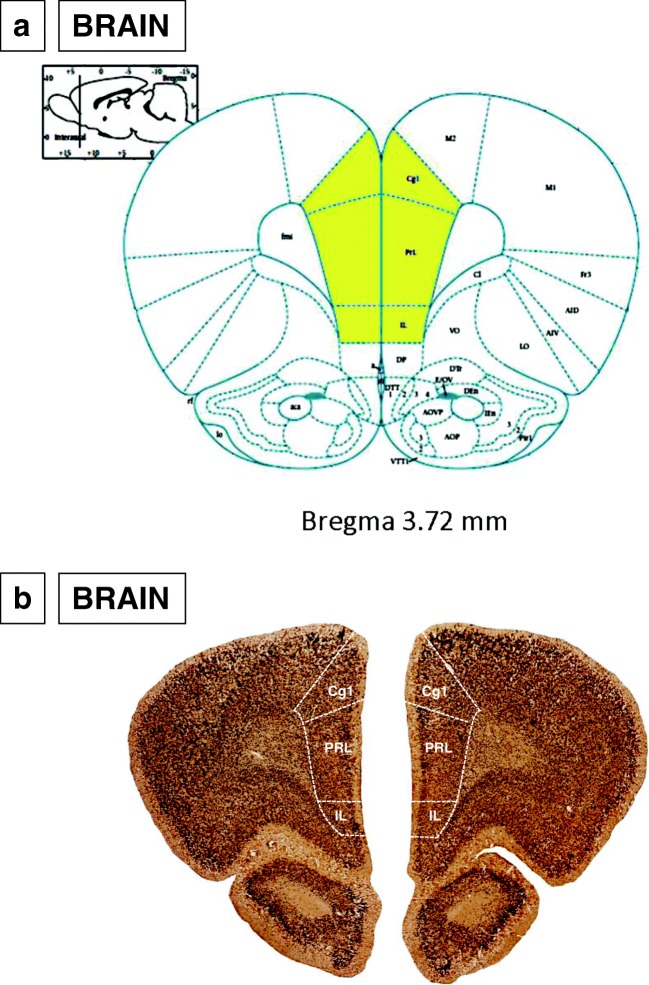
The figure shows the coronal sections (**a**, **b**) of the rat brain with selected areas of the medial prefrontal cortex: cingulated cortex (Cg1), prelimbic cortex (PRL) and infralimbic cortex (IL) from the Paxinos and Watson atlas (**a** Paxinos and Watson 2005) and 5-week-old WKY rat (**b**)

Volumetric measurements of the mPFC in the WKYs and SHRs were done using the image analysis software *Fiji* (Madison, USA) on evenly spaced sections arranged from the rostral to the caudal extent. This region included prelimbic (PRL), cingulate (Cg1) and infralimbic (IL) cortices (Fig. **1**). Every 25th section was stained using the DAB method and antibody against a NeuN protein from the level where the mPFC arrived to the end of it. All of these sections were then digitalised with ×5 magnification using a PathScan Enabler IV Histology Slide Scanner (Praha, Czech Republic). On each digital slice from the bregma 6.12 (Paxinos and Watson 2005), the boundaries of the mPFC (right and left) were outlined by a mouse-driven cursor. The number of sections in the range 28–32 was analysed and these length differences were mostly due to the natural variability among subjects as well as strain and age volumetric differences. The total volumes of the left and right prefrontal cortex were calculated using the formula of DeVito et al. (1989), in which the total volume of a structure (Vo) is the sum of the subvolumes through the structure (Vn). The outlined areas depicting boundaries of the left and right mPFC on the studied sections with the thickness of 250 μm (space between sections) were subvolumes.

To quantify the density of TH and/or D_2_ immunoreactive elements in selected mPFC regions, the sections were analysed using an Olympus BX61 microscope equipped with *cellSens Dimension* image analysing software (Olympus, Tokyo, Japan). The following mPFC regions were tested: PRL, Cg1 and IL. As TH immunoreactivity consisted of nerve fibres, only the Merz grid from the *Fiji* software (Schindelin et al. 2012) was successfully adopted. The D_2_ signal predominated on cell bodies and these cells were manually counted. For each mPFC region in each animal of both rat strains, immunoreactive elements for a particular studied antigen were counted on six evenly spaced sections. In order to test the localisation of the individual PFC regions on the sections, the sections stained with mouse anti-NeuN (pan-neuronal marker) were used. All counts on the single section were made at ×40 magnification using 220 μm × 170 μm regions as the test frames. Depending on the cross-section size of the individual mPFC region, counts were made from either one such field positioned in the centre of the region (and involving 100% of its cross-sectional area) or two to three adjacent non-overlapping fields. All counts made within the test frames in the single mPFC region on the section were averaged. As such mean density value referred only to the area of the test frame, it was always recalculated to show the density of neurons in 1 mm^3^ of the brain tissue. To calculate the mean density of neurons in the whole individual mPFC region in the subject, the means from single sections were averaged. Finally, density values from each mPFC region were averaged in each strain and expressed as means ± standard deviation (SD). All counts were made on coded slides prepared by the first author. To avoid fluorescence fading, each test frame was digitally recorded before counting. Digitalised test frames were then evaluated by two independent experimenters, being blind to the parameters of the studied tissue. The results of these counts showed high inter-rater reliability using a Pearson correlation test (*r* = 0.84, *p* < 0.05).

### Statistical analysis

The statistical differences between groups of data (WKYs vs. SHRs at each matched age) were analysed by one-way ANOVA followed by Tukey test. Moreover, when appropriate, the Mann-Whitney *U* test was conducted. GraphPad Prism 6 software was used to prepare the analyses (GraphPad Software, La Jolla, CA, USA) and *p* < 0.05 was considered to be statistically significant.

## Results

### Pro-inflammatory markers

#### Serum levels of cytokines (Fig. 2a, b)

The serum levels of IL-1β were significantly higher in 5-week-old SHRs than in 5-week-old WKYs (*p* < 0.05). In 10-week-old animals, these levels were significantly reduced in both strains (*p* < 0.001) and reached statistically similar values (*p* > 0.05). In contrast, the serum levels of IL-6 did not differ in 5-week-old SHRs and the age-matched WKYs (*p* > 0.05). In 10-week-old animals, these levels did not change in SHRs (*p* > 0.05) but were significantly reduced in WKYs (*p* < 0.01), causing a significant difference between both strains.

**Fig. 2:**
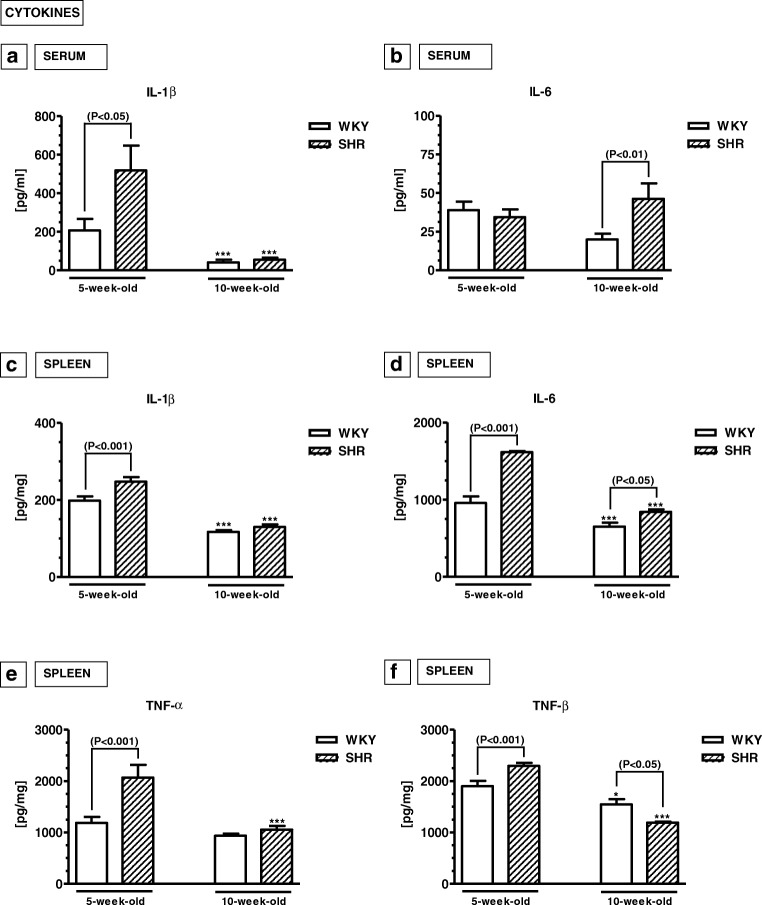
Serum (**a**, **b**) and/or splenic (**c**–**f**) levels of interleukin (IL)-1β (**a**, **c**), IL-6 (**b**, **d**), tumour necrosis factor α (TNF-α; **e**) and transforming growth factor β (TGF-β; **f**). The data are expressed as the mean ± SEM (*n*  =  6). The following statistical levels were applied: *p* < 0.05, *p* < 0.01, *p* < 0.001 indicate differences between the SHR and WKY rats; ^*, ***^ indicate differences (*p* < 0.05; *p* < 0.001) between the juvenile and mature rats of the same strain

#### Splenic levels of cytokines (Fig. 2c–f)

The splenic levels of IL-1β, IL-6, TNF-α and TGF-β were significantly higher in 5-week-old SHRs than in 5-week-old WKYs (*p* < 0.001). In 10-week-old animals of both strains, these levels underwent significant reductions (*p* < 0.05) with diversified effects. For example, the splenic levels of IL-6 were still significantly higher in SHRs (*p* < 0.05). The levels of IL-1β and TNF-α reached similar values in both rat strains (*p* > 0.05). The levels of TGF-β became significantly lower in SHRs than WKYs (*p* < 0.05).

#### Serum levels of chemokines (Fig. 3a–c)

The serum levels of MCP-1, RANTES and IP-10 were significantly higher in 5-week-old SHRs than in 5-week-old WKYs (*p* < 0.04, *p* < 0.02, *p* < 0.001). In 10-week-old animals, these levels did not change in WKYs (*p* > 0.05, except for IP–10), causing similar values in both rat strains.

**Fig. 3:**
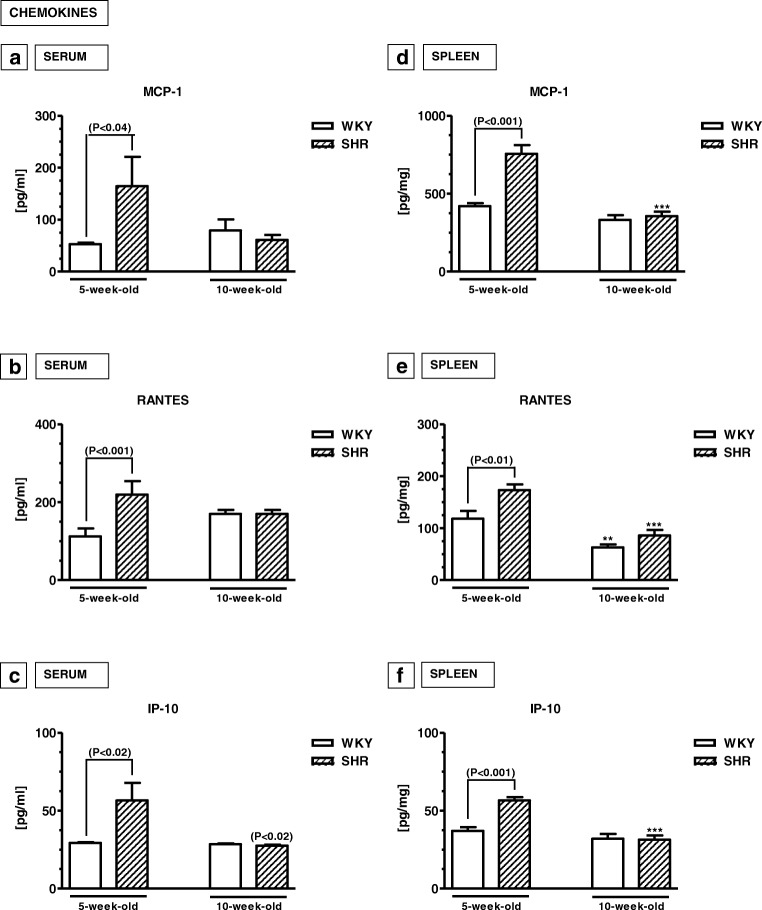
Serum (**a**–**c**) and splenic (**d**–**f**) levels of monocyte chemoattractant protein-1 (MCP-1; **a**, **d**), RANTES (**b**, **e**) and interferon gamma-induced protein 10 (IP-10; **c**, **f**). The data are expressed as the mean ± SEM (*n* =  6). The following statistical levels were applied: *p* < 0.04, *p* < 0.02, *p* < 0.01, *p* < 0.001 indicate differences between the SHR and WKY rats; ^**, ***^ indicate differences (*p* < 0.01; *p* < 0.001) between the juvenile and mature rats of the same strain

#### Splenic levels of chemokines (Fig. 3d–f)

Similar to the serum, the splenic levels of MCP-1, RANTES and IP-10 were significantly higher in 5-week-old SHRs than in age-matched WKYs (*p* < 0.001). In 10-week-old animals, the levels of MCP-1, RANTES and IP-10 levels became similar in both rat strains (*p* > 0.05).

### Oxidative stress markers

**Fig. 4:**
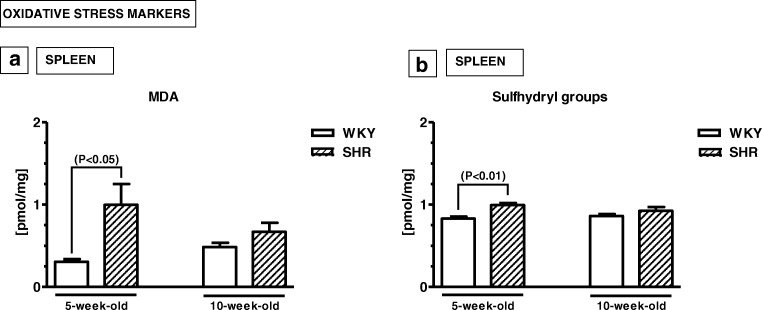
Splenic (**a**, **b**) levels of malondialdehyde (MDA; **a**) and sulfhydryl groups (**b**). The data are expressed as the mean ± SEM (*n* =  6). The following statistical levels were applied: *p* < 0.05, *p* < 0.01 indicate differences between the SHR and WKY rats

The splenic levels of MDA and sulfhydryl groups were significantly higher in 5-week-old SHRs than in 5-week-old WKYs (*p* < 0.05, *p* < 0.01; respectively) (Fig. 4a, b). However, in 10-week-old animals, no significant differences were found between SHRs and WKYs with respect to both of these biomarkers (*p* > 0.05).

### Biomarkers of metabolism

**Fig. 5:**
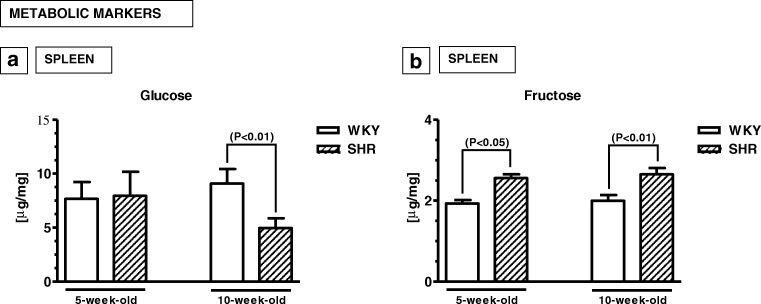
Splenic (**a**, **b**) levels of glucose (**a**) and fructose (**b**). The data are expressed as the mean ± SEM (*n* =  6). The following statistical levels were applied: *p* < 0.05, *p* < 0.01 indicate differences between the SHR and WKY rats

The splenic levels of glucose were similar in 5-week-old animals of both strains (*p* > 0.05), but in 10-week-old animals, it was significantly lower in SHRs than in WKYs (*p* < 0.01) (Fig. 5a). The level of fructose was significantly higher in SHRs compared to WKYs at any age studied (*p* < 0.05, *p* < 0.01) (Fig. 5b).

### Steroid hormones and steroidogenic enzymes

#### Adrenal levels of steroid hormones (Fig. 6a–c)

Adrenal levels of progesterone (P_4_) and cortisol (CT), but not corticosterone (CTT), were significantly elevated in 10-week-old SHRs compared to 10-week-old WKYs (*p* < 0.05, *p* < 0.001; respectively). However, the levels of all these steroid hormones did not differ in 5-week-old animals of both strains (*p* > 0.05).

**Fig. 6:**
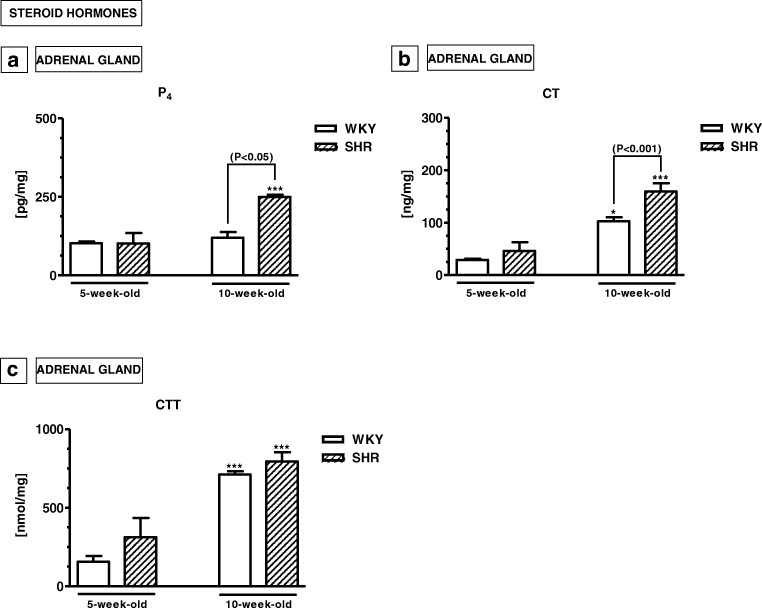
The levels of progesterone (P_4_; **a**), cortisol (CT; **b**) and corticosterone (CTT; **c**) in the adrenal glands. The data are expressed as the mean ± SEM (*n* =  6). The following statistical levels were applied: *p* < 0.05, *p* < 0.001 indicate differences between the SHR and WKY rats; ^*, ***^ indicate differences (*p* < 0.05; *p* < 0.001) between the juvenile and mature rats of the same strain

#### Adrenal levels of steroidogenic enzymes (Fig. 7a–e)

Adrenal levels of 3-beta-hydroxysteroid dehydrogenase/delta(5)-delta(4)isomerase type I (HSD3B1), steroid 21-hydroxylase (CYP21A1) and 11-beta-hydroxylase (CYP11B1) were significantly higher in 5-week-old SHRs than in 5-week-old WKYs (*p* < 0.05, *p* < 0.01). The levels of cytochrome P450 (CYP450) and aldosterone synthase (CYP11B2) did not differ in 5-week-old animals. In 10-week-old rats, the concentrations of all these enzymes were similar in both strains (*p* > 0.05)***.***

**Fig. 7:**
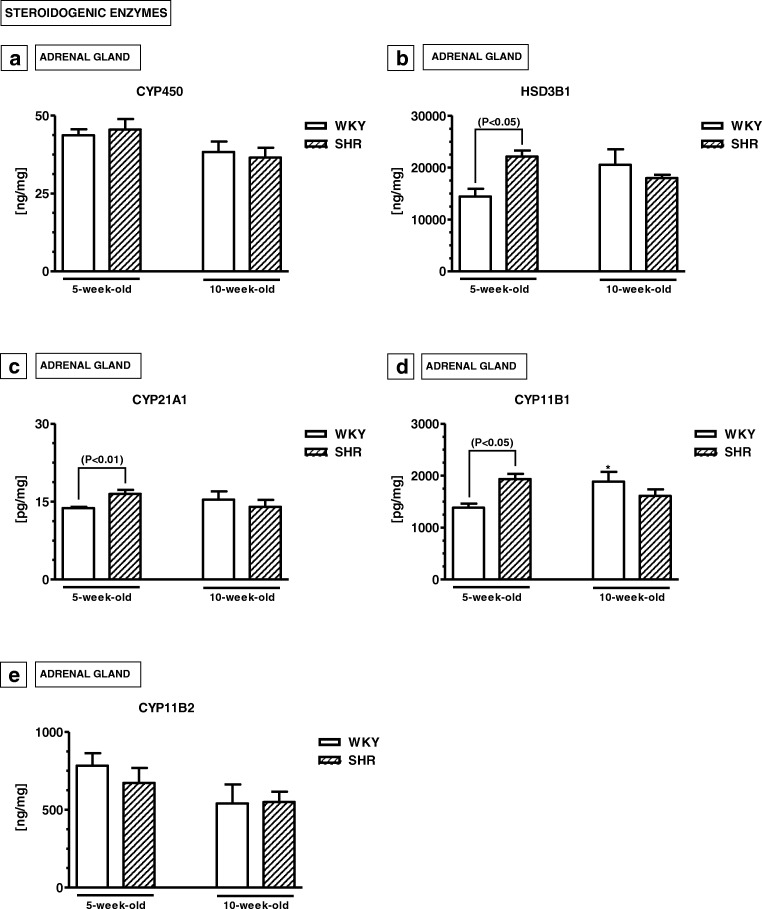
The levels of cytochrome P450 (CYP450; **a**), 3beta-hydroxysteroid dehydrogenase/delta(5)-delta(4)isomerase type I (HSD3B1; **b**), steroid 21-hydroxylase (CYP21A1; **c**), 11-beta-hydroxylase (CYP11B1; **d**) and aldosterone synthase (CYP11B2; **e**) in the adrenal glands. The data are expressed as the mean ± SEM (*n* =  6). The following statistical levels were applied: *p* < 0.05, *p* < 0.01 indicate differences between the SHR and WKY rats; ^*^ indicate differences (*p* < 0.05) between the juvenile and mature rats of the same strain

### Brain alterations

#### Volume of the medial prefrontal cortex (Fig. 8a–d)

The total volumes of the left and right mPFC were significantly reduced in 5-week-old SHRs when compared to 5-week-old WKYs (*p* < 0.001). Bilateral volumetric reductions were observed in all mPFC regions studied, i.e. the PRL, CG1 and IL. However, in the PRL and CG1, these reductions were statistically not significant (*p* > 0.05), but in the IL, they were significant (*p* < 0.01, *p* < 0.001). Interestingly, in 10-week-old animals, the total volumes of the left and right mPFC were significantly larger in SHRs than WKYs (*p* < 0.001). Although the volume of the right IL was still smaller in SHRs (*p* < 0.05), other mPFC regions were similar in both strains or larger in SHRs.

**Fig. 8:**
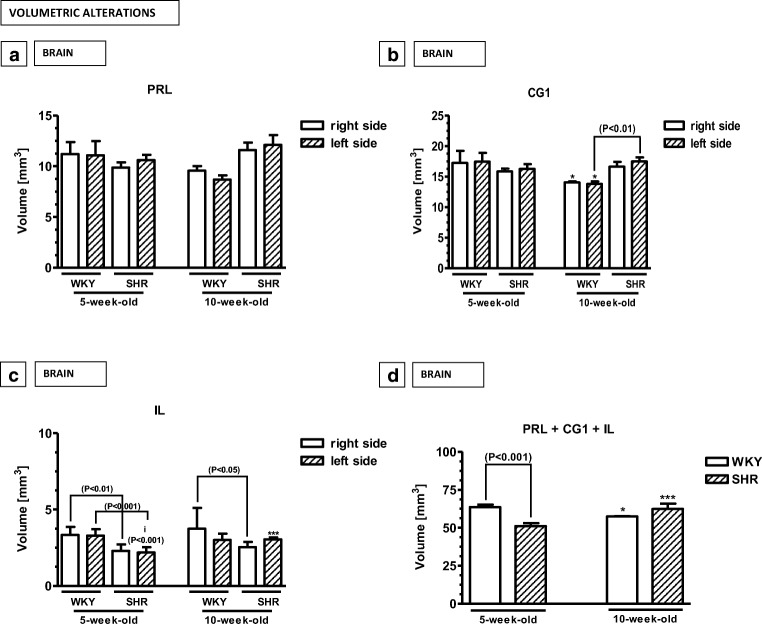
The volume of medial prefrontal cortex (**a**–**d**) and its areas: prelimbic cortex (PRL; **a**), cingulated cortex (CG1; **b**) and infralimbic cortex (IL; **c**). The data are expressed as the mean ± SEM (*n* =  6). The following statistical levels were applied: *p* < 0.05, *p* < 0.01, *p* < 0.001 indicate differences between the SHR and WKY rats; ^*, ***^ indicate differences (*p* < 0.05; *p* < 0.001) between the juvenile and mature rats of the same strain; ^i^ indicates differences (*p* < 0.001) between the right and left hemisphere of 5-week-old SHR rats

#### Dopaminergic markers in the medial prefrontal cortex (Figs. 9a–f, 10a–d and 11a–d)

**Fig. 9:**
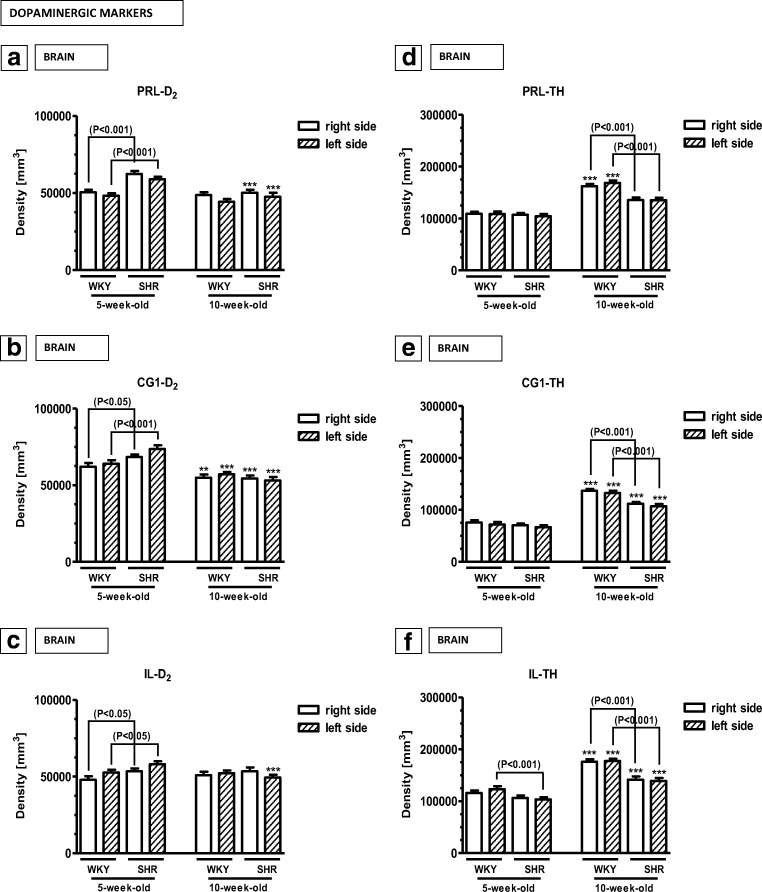
The density [mm^3^] of neurons containing dopamine 2 receptor (D_2_) and nerve fibres containing tyrosine hydroxylase (TH) in the prelimbic cortex (PRL; **a** and **d**, respectively), cingulated cortex (Cg1; **b** and **e**, respectively) and infralimbic cortex (IL; **c** and **f**, respectively). The data are expressed as the mean ± SD (*n* =  6). The following statistical levels were applied: *p* < 0.05, *p* < 0.001 indicate differences between the SHR and WKY rats; ^**, ^^***^ indicates differences (*p* < 0.01, *p* < 0.001) between the juvenile and the mature of the same strain

**Fig. 10:**
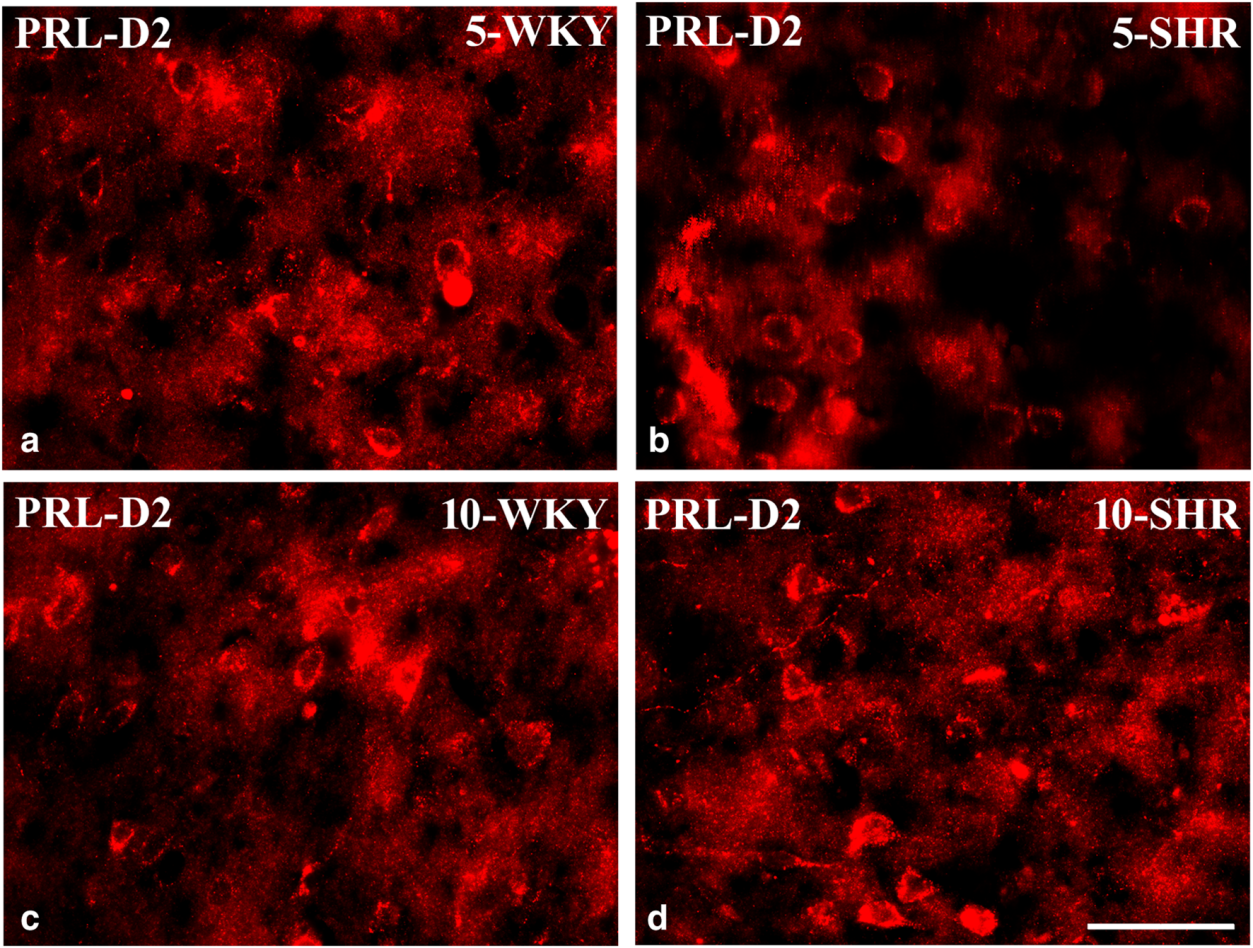
Representative colour photomicrographs illustrating the staining pattern of dopamine 2 receptor (D_2_)-expressing neurons in the prelimbic (PRL) cortex of WKYs (**a**, **c**) and SHRs (**b**, **d**). Note significantly higher density of these cells in 5-week-old SHRs (**b**) when compared to age-matched WKYs (**a**). Note also a lack of significant differences in 10-week-old WKYs (**c**) and SHRs (**d**). Scale bar = 50 μm

**Fig. 11:**
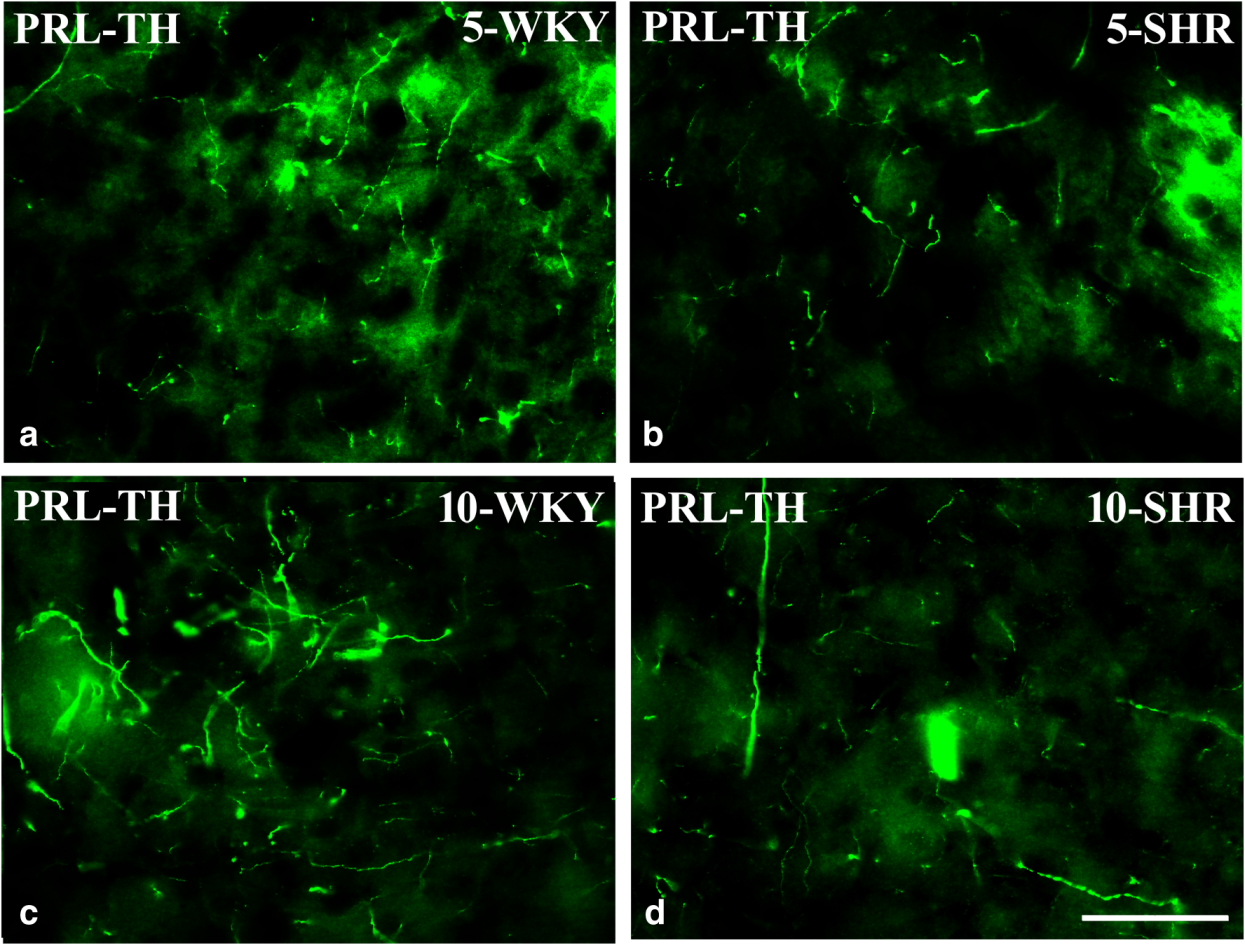
Representative colour photomicrographs illustrating the staining pattern of tyrosine hydroxylase (TH)-expressing nerve fibres in the prelimbic (PRL) cortex of WKYs (**a**, **c**) and SHRs (**b**, **d**). Note a lack of significant differences in 5-week-old WKYs (**a**) and SHRs (**b**). Note also significantly higher density of these fibres in 10-week-old WKYs (**c**) when compared to age-matched SHRs (**d**). Scale bar = 50 μm

The densities of neurons endowed with D_2_ receptors and nerve fibres expressing TH were counted separately in the same mPFC regions. These counts revealed that the density of D_2_-positive cells was significantly elevated in 5-week-old SHRs in the right and left PRL, IL and Cg1 (*p* < 0.05, *p *< 0.001) (Figs. 9a–c and 10a–d). In 10-week-old animals, these values did not differ in both strains in all studied areas (*p* > 0.05). The densities of nerve fibres expressing TH did not differ in 5-week-old rats in the PRL and CG1 (*p* > 0.05), but they vere reduced in the IL (p<0.001). In 10-week-old animals, these values were significantly lower in SHRs in all studied areas (*p* < 0.001) (Figs. 9d–f and 11a–d).

## Discussion

The present results revealed the following facts. (a) Juvenile, but not mature, SHRs have highly elevated serum and/or splenic contents of pro-inflammatory markers, such as cytokines and chemokines which overall may suggest low-grade inflammation in these animals and ADHD patients (Anand et al. 2017). (b) Juvenile, but not mature, SHRs display elevated splenic levels of oxidative stress markers. Interestingly, cytokines may induce and/or increase oxidative stress (Almeida et al. 2011) which was recently reported in ADHD children (Joseph et al. 2015; Sezen et al. 2016). (c) Juvenile and mature SHRs have elevated splenic levels of fructose accompanied by reduced glucose content in mature SHRs. Alterations in both of these metabolic markers may be due to ongoing oxidative stress and/or inflammation. (d) Mature, but not juvenile, SHRs have elevated serum (Kozłowska et al. 2018) and adrenal levels (present results) of steroid hormones, which may be a compensatory (anti-inflammatory) mechanism to treat inflammation and ADHD symptoms (Coutinho and Chapman 2011; Xu et al. 2011). (e) Juvenile and mature SHRs display abnormalities in the mPFC. Juvenile SHRs have significantly reduced volume of the mPFC and elevated expression of D_2_ receptors. In mature SHRs, TH expression is down-regulated. As various factors like pro-inflammatory markers and steroid hormones may directly influence brain development and neurotransmission, they may at least in part cooperate in ADHD pathogenesis (Oades et al. 2010a; Felger and Miller 2012).

As various abnormalities revealed in the present study are especially evident in 5-week-old SHRs and many of them disappear in 10-week-old SHRs, the question is how this phenomenon fits to the rat life cycle and ADHD symptoms. Rats are weaned at an age of 3 weeks and puberty begins at an age of 7–8 weeks, while the approximate weaning age in humans is at an age of 6 months and puberty starts at an age of 11.5 years. In this study, 5-week-old SHRs (approximately equivalent of 7–9-year-old children; Quinn 2005) were characterised by elevated levels of pro-inflammatory (cytokines/chemokines) and oxidative stress markers as well as significantly reduced volume of the mPFC and up-regulation of D_2_ receptors in it. An age of 10 weeks in the rat, when pro-inflammatory and oxidative stress marker levels as well as the mPFC volumes and D_2_ contents for the two strains became quite equal, is the early adolescence. This timing is very similar to clinical findings in ADHD children, for which symptoms become apparent in young school-aged children and hyperactivity improves after puberty. This timing is also consistent with the results of Hsieh and Yang (2008) who investigated the locomotor activity of SHRs and WKYs using a 12-month longitudinal study. These results showed that locomotor activity parameters, such as horizontal activity, total distance and movement time, in SHRs were all the most hyperactive relative to those in WKYs at the age of 5–7 weeks. Since that time, hyperactivity was gradually reduced with age being still present in 10-week-old SHRs and reaching quite similar values in SHRs and WKYs at an age 12 months.

As SHRs are a validated animal model of ADHD (Sagvolden et al*.*^2005^; Sagvolden et al. 2009; Sagvolden and Johansen 2012) and hypertension (Pinto et al. 1998), it is unclear if there is a link between altered serum and/or tissue contents of various markers and ADHD and/or hypertension***.***

### Inflammation and ADHD

#### Cytokine levels

The present results demonstrate that the serum and spleen levels of various cytokines were significantly elevated in 5-week-old SHRs when compared to age-matched WKYs. With age in both rat strains, these levels undergo significant reductions and often reach statistically similar values in 10-week-old animals. The only exception was IL-6, which had similar serum levels in 5-week-old animals of both strains and elevated levels in 10-week-old SHRs (in the spleen, IL-6 was elevated in SHRs at any age studied). Although data concerning cytokine levels in SHRs are scarce, some of them confirm the results of the present study. For example, elevated serum levels of various cytokines were recently reported in 7-week-old SHRs (Chen et al. 2017). On the other hand, the spleen content of IL-6 was significantly higher in 8-week-old SHRs when compared to age-matched Wistar rats (Nakamura et al. 1996). The elevated cytokine levels in 5-week-old SHRs overall suggest a low-grade inflammation in these animals, which coincides with some results in young human ADHD patients. For example, elevated levels of IL-6 and TNF-α in children with ADHD were associated with an intensity of hyperactivity and inattention (Oades et al. 2010b; Allred et al. 2017) although these cytokines do not appear to be involved in ADHD-affected adults (Corominas-Roso et al. 2017). Interestingly, elevated levels of IL-6 and TNF-α were noted in the present study in juvenile SHRs. An important role of IL-6 and TNF-α in the etiopathogenesis of ADHD may explain significant connections between IL-6 and TNF-α gene polymorphism and ADHD (Drtilkova et al. 2008). In addition, it was reported that a large percentage of children with ADHD had detectable IL-2, IFN-γ, TNF-β, IL-5 and IL-10 concentrations in the cerebrospinal fluid (Mittleman et al. 1997). This fact matches well with evidence that inflammatory cytokines may interfere with the maturation of the PFC in ADHD-affected individuals (Buske-Kirschbaum et al. 2013). Moreover, there are many studies reporting that administration of IL-1β and IL-6 in rodents reduced the level of dopamine in the brain (Zalcman et al. 1994; Anisman et al. 1996), similar to ADHD patients (Blum et al. 2008). Interestingly, elevated levels of IL-6 accompanied by a reduction in the density of TH-positive fibres in the mPFC were also observed in 10-week-old SHRs (present study). It should be noted, however, that there is also opposite data, making the picture much more obscure. For example, Oades et al. (2010a) reported that serum levels of IL-1β were lower in ADHD children when compared to the control counterparts. In addition, elevated serum levels of IL-6 were reported by Donfrancesco et al. (2016) in ADHD children, but not adults. Corominas-Roso et al. (2017) did not observe significant differences in the serum IL-6 content between adult ADHD patients and controls. These discrepancies may be partially due to the differences between ages, samples and the different sensitivity and specificity between ELISA kits. Another important fact is that altered cytokine contents in SHRs may play important roles in both ADHD and hypertension (Conrad et al. 1995; Schiffrin 2014). For example, elevated levels of IL-6 and/or TNF-β were associated with myocardial fibrosis and hypertrophy of the left ventricle (Lijnen et al. 2003; Kurdi et al. 2005; Meléndez et al. 2010).

#### Chemokine levels

The results of the present study showed that the serum and splenic concentrations of chemokines such as MCP-1, RANTES and IP-10 were also significantly elevated in 5-week-old SHRs when compared to age-matched WKYs and 10-week-old animals of both strains. For this reason, the pattern of chemokine contents in juvenile SHRs is quite consistent with that of cytokine contents and corroborates the impression of a low-grade inflammation in these animals. Unfortunately, it is difficult to compare these results with previous studies because in the available literature there is a lack of detailed data on the level of chemokines in SHRs and/or ADHD children/adults. To date, it was only reported that diabetic SHRs had an elevated serum level of RANTES (Mason et al. 2015). It is generally known that in the physiological state, chemokines and their receptors are responsible for proper communication between neurons and inflammatory cells (Bajetto et al. 2001). Moreover, they are involved in neuronal death and neurodegenerative diseases (Cartier et al. 2005). However, data concerning the role of chemokines in the ADHD pathogenesis is lacking. To date, it has only been reported that top-quartile concentrations of RANTES may increase the risk of ADHD symptoms (Allred et al. 2017). This hypothesis may partly be supported by study in a mouse model of Parkinson disease where peripheral administration of blocking antibodies against RANTES reduced the infiltration of CD4^+^ and CD8^+^ T cells into the substantia nigra and prevented the loss of dopaminergic neurons (Chandra et al. 2016). It is worth noting that secretion of RANTES may be induced by TNF-α and modulated by glucocorticoids (Ammit et al. 2002) and the levels of both of these factors were significantly altered in SHRs. For IP-10, it was only reported that increased levels of this chemokine were observed in patients with acute inflammatory demyelinating polyradiculoneuropathy (Kieseier et al. 2002). Interestingly, a recent study found a strong association between paediatric demyelinating diseases of the central nervous system and various psychiatric disorders, including ADHD (Pakpoor et al. 2018). In the case of MCP-1, it is known that it is expressed in the cerebral cortex (Banisadr et al. 2005) which is abnormal in ADHD children (Wolosin et al. 2009). Additionally, prolonged exposure of dopaminergic neurons in the rat substantia nigra slices to MCP-1 increases dopamine release (Guyon et al. 2009). However, the participation of chemokines and their receptors in hypertension was also postulated (Martynowicz et al. 2014; Rudemiller and Crowley 2017) although this issue is still not fully understood. For example, the overexpression of MCP-1 was observed in hypertensive rats and human patients and this chemokine was proposed as a marker of organ damage in hypertensive heart disease (Zhuo 2004; Tucci et al. 2006). In turn, RANTES induced elevated expression of IL-10 and, in this way, exerted antihypertensive effects of IL-10 in the vascular smooth muscle cells of SHRs (Kim et al. 2015).

### Oxidative stress and ADHD

As the levels of pro-inflammatory cytokines were also correlated with the severity of symptoms in the ADHD children (Oades et al. 2010b), one of the underlying mechanisms of enhanced inflammation in children and juvenile SHRs could be a stress-related immune response. Interestingly, the present results demonstrate that the levels of MDA and free sulfhydryl groups in the spleen were significantly higher in 5-week-old SHRs than in age-matched WKYs. The levels of MDA were also higher in 10-week-old SHRs, but the difference was not significant. Thus, the present results are congruent with previous studies reporting higher levels of MDA in the serum of young and adult ADHD patients (Bulut et al. 2007; Ashour et al. 2016) and mature SHRs (Duarte et al. 2001). However, they are contrary to the findings of Oztop et al. (2012) who reported lower MDA levels in ADHD children. For sulfhydryl groups, the data are very limited. It has only been reported that sulfhydryl levels were significantly higher in children and adolescent ADHD patients than in control patients (Guney et al. 2015). A direct relationship between oxidative stress and ADHD was reported by Bulut et al. (2007), Oztop et al. (2012) and Sezen et al. (2016). Moreover, Verlaet et al. (2018) suggested that chronic inflammation and oxidative stress can lead to ADHD symptoms, for example, by chronic T-cell–mediated neuroinflammation, as well as by neuronal oxidative damage and loss of normal cerebral functions. Additionally, oxidative stress may influence dopamine synthesis, neuronal cell migration and plasticity (Verlaet et al. 2018) which are disrupted in ADHD patients (Blum et al. 2008; Asherson and Gurling 2012). It is worth noting that some previous studies also demonstrated a relationship between oxidative stress and hypertension (Armas-Padilla et al. 2007; Ahmad et al. 2013) which finally led to significant renal damage in rats (Manning Jr et al. 2005).

### Biomarkers of metabolism and ADHD

According to the present results, the level of fructose in the spleen was significantly higher in SHRs than WKYs at any age studied. In contrast, glucose level was significantly decreased in 10-week-old SHRs when compared to 10-week-old WKYs. There is currently no detailed data on the levels of fructose and glucose in the spleen of SHRs and/or WKYs. However, it is possible that low glucose levels in 10-week-old SHRs may be associated with high levels of fructose (Huang et al. 2004). The role of both of these carbohydrates in ADHD pathogenesis is obscure. There is evidence that a high level of fructose in the spleen (and probably in the serum) might disrupt energy metabolism and brain plasticity, as was suggested in the traumatic brain injury patients (Agrawal et al. 2016). Glucose may have effects on mesolimbic dopamine signalling (Johnson et al. 2011). For example, a high dose glucose treatment for 12 h followed by 12 h food deprivation resulted in reduction of D_2_ binding in the dorsal striatum and it increased dopamine transporter binding in the midbrain (Colantuoni et al. 2001). As far as hypertension is concerned, it was reported that uncontrolled metabolism of fructose leads to hypertension and metabolic syndrome (Khitan and Kim 2013). Moreover, treatment with 10% fructose in drinking water induced hypertension in Wistar rats, which was associated with elevated levels of plasma insulin, glucose and triglycerides (Dai and McNeill 1995). Interestingly, in the present study, the level of glucose in 5-week-old SHRs and WKYs was similar.

### Steroid hormones and steroidogenic enzymes and ADHD

The pattern of steroid hormone contents seems to be quite opposite to that of pro-inflammatory and oxidative stress markers. For example, the present results indicated that the adrenal levels of P_4_, CT and CTT did not differ in 5-week-old SHRs and WKYs. On the other hand, in 10-week-old animals, P_4_ and CT levels were significantly higher in SHRs. Interestingly, an elevated content of steroidogenic enzymes such as HSD3B1, CYP21A1 and CYP11B1 was only observed in the adrenal gland of 5-week-old SHRs. In mature animals, these levels were similar in both rat strains. It is difficult to explain these phenomena, because in the available literature there is a lack of detailed data on this topic. It was only reported that serum levels of P_4_, CT and CTT were also significantly elevated in the 10-week-old SHRs when compared to age-matched WKYs, and these levels did not differ in WKYs at any age studied (Kozłowska et al. 2018)*.* The role of steroid hormones in ADHD pathogenesis was postulated. According to previous studies, steroid hormones may intensify or weaken ADHD symptoms (Martel et al. 2009; Gaysina et al. 2012; Torregrossa et al. 2012; Liu and Wang 2015; Roberts 2016). Moreover, they may also modulate cortical and/or striatal dopamine concentrations directly and/or indirectly by modulating the secretion of cytokines and chemokines (Xiao and Becker 1994; Batuman et al. 1995; Elenkov et al. 1996; Ramírez et al. 1996; Franchimont et al. 1999; Galon et al. 2002; Fukakusa et al. 2005; Wingett et al. 2011; Martino et al. 2012; Chen et al. 2017a). Interestingly, the present study revealed inverse correlation between inflammatory markers and steroid hormone contents in SHRs. Thus, the reduction in cytokine, chemokine and oxidative stress marker contents in mature SHRs might be associated with immunosuppression caused by high P_4_ and CT levels (Coutinho and Chapman 2011). It should be noted, however, that steroid hormones may also be implicated in hypertension, in both rodents and humans (Yagil et al. 1996; Whitworth et al. 1998). For example, glucocorticoids may raise blood pressure in humans (Kelly et al. 1998; Whitworth et al. 2005).

### Brain alterations and ADHD

#### Morphometry of the mPFC

The results of the present study revealed some morphological abnormalities in the mPFC of SHRs. For example, the volumes of the right and left IL were significantly reduced in 5-week-old SHRs when compared to age-matched WKYs. The reduced volume of the IL persisted in 10-week-old SHRs, but only in the right hemisphere. Although there are no volumetric measurements of the mPFC in SHRs, reduced thickness of the frontal cortex in SHRs was previously reported (Tajima et al. 1993). Moreover, any of the studies measuring at least one compartment of the PFC reported smaller volumes in ADHD patients. Some studies have reported ADHD-related reductions in the mPFC (Castellanos and Proal 2009), which coincides with the present results. Other studies have indicated reductions in the dorsolateral regions of the PFC (Hynd et al. 1990; Castellanos et al. 1996, 2002; Filipek et al. 1997; Kates et al. 2002; Mostofsky et al. 2002; Hill et al. 2003; Durston et al. 2004). The inconsistency in volumetric studies in human ADHD patients is probably due to the small number of subjects, the influence of medications, comorbidities or gender and has not addressed potentially important sources of heterogeneity, such as a family history of ADHD, DSM subtype or perinatal complications (Seidman et al. 2005). Broadly, the developmental progression of ADHD symptoms parallels the emergence of control processes mediated by the maturation of the PFC (Vaidya 2012). Thus, age-inappropriate levels of hyperactivity/impulsivity and inattention could reflect a maturational course of the PFC that is atypical or typical but delayed by a few years in ADHD children (Vaidya 2012). Moreover, typical development includes an overall increase in cortical volume prior to puberty followed by reductions in adolescence (Giedd et al. 1999). However, it seems that in ADHD-affected individuals, there is a developmental shift of both these processes which is reflected by the present results. Volume reductions in the mPFC were also primarily observed in the present study in juvenile SHRs when the contents of cytokines, chemokines and oxidative stress markers were very high. This finding matches recent evidence that significantly reduced cortical grey matter volumes were found in cases with schizophrenia and ‘high inflammation’ status relative to schizophrenia cases with ‘low inflammation’ status in the PFC (Zhang et al. 2016). The present results also indicated that in 10-week-old SHRs, volume reduction of the mPFC persisted, but only in the right hemisphere. This observation might confirm atypical brain laterality observed earlier in adult patients with ADHD (right hemisphere deficit) (Mohamed et al. 2015). Moreover, a recent review of fNIRS studies has reported that right hemisphere asymmetry in atypical neuronal function was found in children with ADHD (Doi and Shinohara 2017). Another longitudinal study has shown that healthy children progressively developed normal lateralisation of the right frontal cortex and left occipital cortex during development, although this normal lateralisation would be disrupted for ADHD children (Shaw et al. 2009). In addition, the asymmetry of the hemisphere has also been found in the frontostriatal white matter in ADHD children (Silk et al. 2016). Thus, it seems that lateralisation is quite an important factor in morphometric studies on ADHD patients and various inconsistencies in the literature may reflect the influence of such asymmetries.

#### Dopaminergic markers in the mPFC

The present results also demonstrate increased density of neurons expressing D_2_ in 5-week-old SHRs and reduced density of TH-positive fibres in 10-week-old SHRs. Although some studies suggest no differences in D_1_ and D_2_ expression in SHRs compared to WKYs (Fuller et al. 1983; Van den Buuse et al. 1992; Linthorst et al. 1993), many others indicate up-regulation of D_1_ and D_2_ receptors in several brain areas of SHRs, including the frontal cortex, nucleus accumbens and striatum (Chiu et al. 1982, 1984; Kirouac and Ganguly 1993; Lim et al. 1998; Sadile 2000; Papa et al. 2002). The up-regulation of D_2_ observed in the present study in juvenile SHRs coincides well with recent evidence that astrocytic D_2_ modulate innate immunity and, when activated, usually suppress neuroinflammation in the central nervous system through a αB-crystallin-dependent mechanism (Shao et al. 2013). Thus, up-regulation of D_2_ in juvenile SHRs when the contents of cytokines, chemokines and oxidative stress markers are very high might be an anti-inflammatory effect. Moreover, the up-regulation of D_2_ observed in the present study also matches the fact that these receptors seem to mediate both hyperactivity and amphetamine responses observed in ADHD-affected human patients and animals (Fan et al. 2010). For example, in coloboma mice, which are a mice model of ADHD, targeted deletion of the D_2_ (but not the D_3_ or D_4_) dopamine receptor eliminated the hyperactivity. Amphetamine treatment in coloboma mice and ADHD human patients has similar effects on hyperactivity (Fan et al. 2010). The D_2_ dopamine receptor-selective antagonist L-741,626, but not D_3_ or D_4_ dopamine receptor-selective antagonists, blocked the amphetamine-induced reduction in locomotor activity. On the other hand, ropinirole and pergolide (D_2_ agonists) improve the symptoms of ADHD in children treated with these agonists for restless leg syndrome (Walters et al. 2000; Konofal et al. 2005). Likewise, pergolide not only reduces tics in children, but it also improves ADHD (Gilbert et al. 2003). It should be noted that the family of D_1_-like dopamine receptors does not contribute to an amphetamine-induced decrease in hyperactivity (Fan and Hess 2007). Thus, the D_2_ dopamine receptor subtype, specifically, seems to mediate both hyperactivity and the response to amphetamine, suggesting a specific target for novel therapeutics in ADHD. It is worth mentioning here that although the effects of various therapeutics (methylphenidate, amphetamine, etc.) on dopamine neurotransmission and ADHD symptoms have been widely studied (Andersen et al. 2002; Yang et al. 2003), little is known on how these drugs target the immune and endocrine systems which are discussed in the present study. It is unlikely that the immune/endocrine systems are not at least partially influenced by these drugs. The reduced density of TH-positive fibres in SHRs observed in the present study was also reported previously (Leo et al. 2003; Viggiano et al. 2004). As tyrosine hydroxylase is the rate-limiting enzyme of catecholamine biosynthesis, both mechanisms, i.e. D_2_ up-regulation and TH down-regulation, actually lead to reduced dopamine activity in the PFC, which produces hyperactivity in animals (Simon 1981).

## Conclusions

These results suggest an ongoing inflammatory process accompanied by oxidative stress in juvenile SHRs, which can affect brain morphology and the brain dopamine level and, in this way, participate in ADHD pathogenesis (Zalcman et al. 1994; Anisman et al. 1996; Oades et al. 2010a; b; Felger and Miller 2012). Moreover, elevated levels of progesterone and glucocorticoids in adult SHRs may be a compensatory effect associated with a reduction of inflammation and ADHD symptoms (Coutinho and Chapman 2011; Xu et al. 2011; Torregrossa et al. 2012) observed in these animals. However, it should be noted that both of these processes may also lead to hypertension (Whitworth et al. 1998; Ghanem and Movahed 2007).
